# Trends in Pharmacist‐Prescribed Dispensing Records of HIV Pre‐Exposure (2020–2025) and Post‐Exposure Prophylaxis (2020–2024) in Brazil: A Time Series Analysis

**DOI:** 10.1002/pds.70422

**Published:** 2026-07-03

**Authors:** Gustavo Magno Baldin Tiguman, Dyego Carlos Souza Anacleto de Araújo, Patricia Melo Aguiar

**Affiliations:** ^1^ Department of Pharmacy, Faculty of Pharmaceutical Sciences University of São Paulo São Paulo Brazil; ^2^ Department of Pharmaceutical Sciences Federal University of Espírito Santo Espírito Santo Brazil

**Keywords:** Brazil, HIV prevention, pharmacists, post‐exposure prophylaxis, pre‐exposure prophylaxis, prescriptions, time series studies

## Abstract

**Purpose:**

Pharmacist prescribing of HIV pre‐exposure (PrEP) and post‐exposure prophylaxis (PEP) is an emerging strategy to expand access to HIV prevention, but real‐world evidence on its implementation remains limited. We assessed national and regional trends in pharmacist‐prescribed dispensing records of PrEP (2020–2025) and PEP (2020–2024) in Brazil.

**Methods:**

Nationwide time series analysis using databases from the Ministry of Health. The primary outcome was pharmacist‐prescribed PrEP and PEP dispensing records per 100 000 adults per year. Joinpoint regression was used to estimate average annual percent change (AAPC) and 95% confidence intervals (95% CIs).

**Results:**

Pharmacists prescribed 27 126 (2.80%) of 969 536 PrEP records and 13 073 (1.97%) of 664 000 PEP records. The frequency of pharmacist‐prescribed records in relation to all prescribers increased (PrEP: 0.22% to 5.77%; PEP: 0.12% to 4.59%). National pharmacist‐prescribed PrEP dispensing increased from 0.10 to 6.24 per 100 000 adults (AAPC: 120.80; 95% CI 71.96 to 189.84; *p* < 0.001), while pharmacist‐prescribed PEP dispensing increased from 0.07 to 4.93 per 100 000 adults (AAPC: 166.62; 95% CI 68.66 to 313.26; *p* < 0.001). The Southeast had the highest absolute rates for both strategies. Significant upward trends were observed in all five regions for PrEP, and in the Southeast, Northeast, and Center‐West for PEP. The national PrEP rate stabilized between 2024 and 2025.

**Conclusions:**

Pharmacist prescribing of HIV prophylactic medications significantly increased in Brazil since 2020 and may potentially represent an important mechanism to broaden access to HIV prevention. However, regional differences may underscore the need for targeted policy interventions to address geographic implementation gaps.

## Key Points

- Real‐world study of trends in pharmacist prescribing of PrEP (2020–2025) and PEP (2020–2024) in Brazil.
- Pharmacists were prescribers for 2.80% of all PrEP dispensing records and 1.96% of PEP records.
- Pharmacist‐prescribed dispensing records grew exponentially in Brazil for PrEP and PEP.
- The Southeast had the highest absolute dispensing rates for both strategies.
- Upward trends were observed in all five regions for PrEP, and in the Southeast, Northeast, and Center‐West for PEP.

## Executive Summary

Allowing pharmacists to prescribe HIV prophylactic medications—known as pre‐exposure prophylaxis (PrEP) and post‐exposure prophylaxis (PEP)—is a strategy to increase access to HIV prevention. In Brazil, pharmacists are authorized to do this within specialized public HIV services, following national clinical protocols. This study examined trends in pharmacist‐prescribed dispensing records of PrEP (2020–2025) and PEP (2020–2024) in Brazil. Using national health databases, we found that pharmacist‐prescribed dispensing records grew rapidly over this period. When looking at their overall share of the healthcare workload, the proportion of pharmacist‐prescribed PrEP dispensing records increased from 0.22% to 5.77%, while their share of pharmacist‐prescribed PEP records grew from 0.12% to 4.59%. The number of these records per 100 000 adults also increased significantly across the country. Pharmacist‐prescribed PrEP dispensing records rose from 0.10 to 6.24 per 100 000 adults, whereas pharmacist‐prescribed PEP records increased from 0.07 to 4.93 per 100 000 adults. The Southeast region of Brazil had the highest dispensing rates. These findings show that pharmacists are playing an increasingly important role in broadening access to HIV prevention in Brazil. However, because the growth is uneven across different parts of the country, targeted health policies are needed to address these regional gaps and ensure equal access.

## 1 Introduction

HIV pre‐exposure prophylaxis (PrEP) and post‐exposure prophylaxis (PEP) are highly effective biomedical strategies for preventing HIV acquisition and constitute central components of global HIV prevention policies [1, 2]. Daily oral PrEP reduces the risk of sexual HIV transmission by more than 90% when adherence is adequate, while PEP remains an essential emergency intervention following potential exposure [1, 2]. Expanding access to these interventions is a priority for achieving the Joint United Nations Programme on HIV/AIDS (UNAIDS) targets aimed at ending the HIV epidemic as a public health threat [3, 4].

Pharmacists have increasingly assumed expanded clinical roles worldwide, including prescribing authority for HIV prophylaxis—under both dependent and independent prescribing models—as a strategy to reduce structural barriers such as limited physician availability, stigma, and delays in care access [5]. Pharmacist‐led PrEP and PEP services have demonstrated feasibility, safety, and high patient acceptability in multiple health systems, contributing to improved linkage to prevention services and medication initiation [6, 7, 8, 9]. In Brazil, HIV prophylaxis is predominantly delivered through the Brazilian Unified Health System (*Sistema Único de Saúde*—SUS) [10]. Pharmacist prescribing of HIV prophylaxis was first operationalized in 2020 through local clinical protocols pioneered by the São Paulo State Department of Health [11]. Over the following years, this expanded scope of practice navigated significant regulatory instability, experiencing alternating periods of authorization and suspension by authorities, but was ultimately consolidated and granted nationwide approval in 2023, following endorsements from the Federal Council of Pharmacy and the Brazilian Ministry of Health [11, 12, 13, 14, 15].

Despite these regulatory advances, national evidence describing how pharmacist prescribing has evolved after implementation remains limited. Understanding temporal trends and regional patterns is critical to evaluate workforce integration and inform future public health strategies. Therefore, this study aimed to assess trends in pharmacist‐prescribed dispensing records of PrEP (2020–2025) and PEP (2020–2024) across Brazilian regions using national public data from the Brazilian Ministry of Health.

## 2 Methods

### 2.1 Study Design

This was a time series analysis with joinpoint regression of pharmacist‐prescribed dispensing records of HIV PrEP (2020–2025) and PEP (2020–2024) in Brazil. The reporting of the study followed the recommendations of the Strengthening the Reporting of Observational Studies in Epidemiology Checklist (STROBE) for observational studies and the REporting of studies Conducted using Observational Routinely‐collected health Data (RECORD) extension [16, 17].

### 2.2 Setting

Brazil provides universal and free access to HIV prevention, testing, and treatment through the SUS, a publicly funded national healthcare system organized across primary, specialized, and hospital levels of care [10]. HIV prevention policies are coordinated nationally by the Ministry of Health and implemented through a network of specialized HIV and sexually transmitted infection services, testing and counseling centers, outpatient infectious disease clinics, and exposure reference units responsible for delivering prevention and care interventions [10, 18, 19].

The Brazilian HIV response adopts a combined prevention framework integrating behavioral, biomedical, and structural interventions aligned with World Health Organization recommendations [4]. Biomedical strategies currently incorporated within the SUS include universal antiretroviral therapy, treatment as prevention, PrEP and PEP, HIV testing and self‐testing, and condom distribution programs [10, 19, 20]. Oral daily PrEP was incorporated into the SUS in 2017 and is primarily delivered through specialized HIV services targeting populations at increased risk of HIV acquisition as a continuing regimen (daily or on‐demand) [19, 21]. During the study period, oral PrEP within the SUS was based on tenofovir disoproxil fumarate combined with emtricitabine (TDF/FTC) [22]. PEP is available nationwide since 2013; treatment must be initiated within 72 h following potential HIV exposure and taken for 28 days, being provided through emergency services and specialized HIV reference units [19, 23]. The first‐line SUS regimen during the study period combined tenofovir disoproxil fumarate and lamivudine with dolutegravir (TDF/3TC + DTG), with alternatives where contraindicated [24].

Brazil is a continental, highly heterogeneous country in which HIV prevention infrastructure, the health workforce, and specialized services have historically concentrated in the wealthier, more urbanized South and Southeast, while the North and Center‐West face greater geographic and socioeconomic barriers to healthcare access [18, 25]. These regional disparities in income, urbanization, and service density may influence the uptake of new prescribing models.

### 2.3 Pharmacist‐Prescribed PrEP/PEP in Brazil

Unlike certain healthcare systems where community pharmacies independently deliver HIV prophylaxis, prescribing of PrEP and PEP in Brazil occurs predominantly within specialized SUS HIV services operating under standardized national clinical protocols through dependent (collaborative, protocol‐based) prescribing models rather than fully independent prescribing [12, 15]. These services function through multidisciplinary teams including physicians, nurses, pharmacists, and other healthcare professionals responsible for clinical assessment, testing, prescribing, medication dispensing, counseling, and follow‐up [10].

Pharmacists prescribe PrEP and PEP within their professional scope as defined by the Federal Council of Pharmacy regulations on clinical and prescribing activities, and act in accordance with the national PrEP/PEP clinical protocols [26, 27]. Pharmacist prescribing of HIV prophylactic medications was first operationalized in 2020 through a municipal protocol of the São Paulo Department of Health, progressively endorsed by the Federal Council of Pharmacy in 2022 and consolidated nationally by the Ministry of Health in 2023 [11, 12, 13, 14, 15]. The authorization represented a task‐sharing strategy intended to expand timely access to HIV prevention while maintaining integration within established national care pathways [11, 12, 13, 14, 15]. Service‐level qualification in the applicable clinical protocols is expected before pharmacists prescribe within a given service.

**FIGURE 1:**
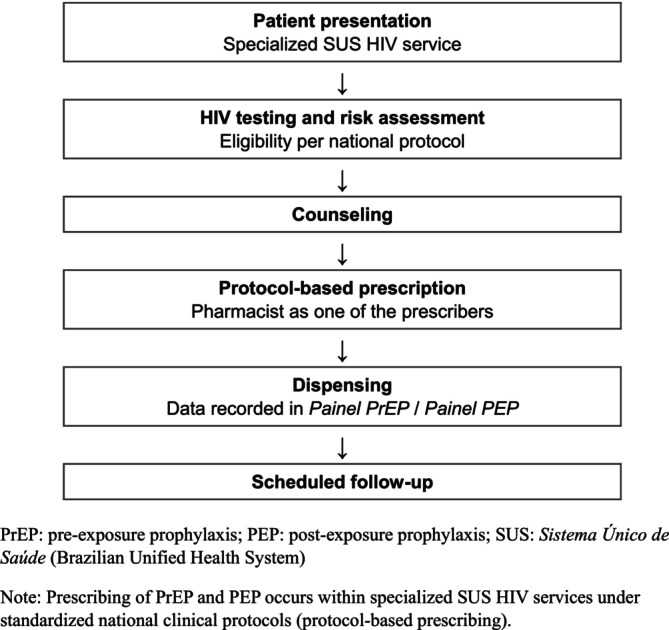
Workflow of PrEP and PEP prescribing within specialized SUS HIV services in Brazil.

The typical process workflow of prescribing comprises patient presentation, HIV testing and risk assessment, counseling, protocol‐based prescription, medication dispensing, and scheduled follow‐up [22, 24] (Figure 1). Prescribing occurs predominantly within public‐sector specialized services.

The national protocols do not impose a fixed numerical cap on the number of prescriptions a qualified prescriber may issue; prescribing is instead bounded by the clinical protocol and the regimen structure [22, 24]. Because PEP is a single time‐limited course whereas PrEP is a continuing regimen with scheduled re‐dispensing, this distinction is central to interpreting the dispensing‐record outcome.

### 2.4 Sample, Variables and Data Sources

The primary outcome was the number of pharmacist‐prescribed dispensing records of HIV prophylactic medications per 100 000 adults per year. A dispensing record was defined as a single registered dispensing event for which a pharmacist is the recorded prescriber; it does not necessarily correspond to a unique individual. For PEP, treatment is a single 28‐day course; therefore, each record approximates one episode/individual; for PrEP, a continuing regimen, repeated re‐dispensing to the same individual is expected, so records represent dispensing events rather than unique users. Additional analyses stratified by Brazilian region (Center‐West, Northeast, North, Southeast, South) were also conducted.

Data were obtained from the “Painel PrEP” and “Painel PEP” public databases [28, 29]. Both monitoring panels were developed and published by the Brazilian Ministry of Health with the aim of disseminating information on the dispensing and the use of HIV prophylactic medications within the SUS. The available datasets contained dispensing data retrieved from Medication Dispensing Units and Exposure Reference Units between 2018 and 2025 for PrEP and between 2018 and 2024 for PEP [28, 29].

The PrEP datasets included information about each PrEP dispensed within the SUS, including the name of the Medication Dispensing Unit, the date of dispensing, the location (state and region), the PrEP modality (daily PrEP, on demand), the type of healthcare service (public, private), treatment range between first and last dispensing of > 1 year (yes or no, considering the difference between the dates of first and last PrEP dispensing), and the type of prescriber (pharmacist, physician, nurse, dentist) [28]. All records available in the datasets included data on the type of prescriber.

The “Painel PEP” database contained additional patient information since treatment duration is limited to 28 days after HIV exposure, meaning that each dispensing record corresponds to an individual patient. In addition to the name of the Medication Dispensing Unit, the date of dispensing and the location (state and region), the PEP datasets also included information on patient age group (0–14, 15–24, 25–39, 40–59, ≥ 60 years old), self‐declared race/skin color (White/Asian, Indigenous, Brown [Brazilian mixed‐race], Black), years of educational background (≤ 3, 4–7, 8–11, ≥ 12 years), type of HIV exposure (accident with biological material, consented sexual exposure, sexual violence), and type of prescriber (pharmacist, physician, nurse, dentist) [29]. Similarly to the PrEP database, all records included the type of prescriber.

The total number of records was divided by the population estimates for adults ≥ 18 years old for each Brazilian region [30], resulting in the number of pharmacist‐prescribed dispensing registries of HIV prophylactic medications per 100 000 adult inhabitants per year. We used the general adult population as a standardized, transparent, and reproducible denominator that allows comparison of dispensing activity across regions of very different size, in the absence of reliable region‐specific estimates of the population eligible for PrEP/PEP. Because PrEP and PEP are targeted interventions, this denominator describes population‐level dispensing activity rather than coverage or access among those at risk.

The panels provide location at the level of state and region and the name of the dispensing/exposure unit, but do not include a validated municipal urban/rural classification field. We therefore did not conduct urban/rural stratified analyses; instead, analyses were stratified by macro‐region, which captures a recognized axis of the infrastructure and urbanization gradient in Brazil [28, 29].

The study sample comprised all dispensing records attributed to a pharmacist prescriber between 2020 and 2025 (PrEP) and between 2020 and 2024 (PEP). Since pharmacist prescribing of PrEP and PEP was first operationalized in 2020 in the country through local clinical protocols [12], no records of pharmacist prescriptions of HIV prophylactic medications were registered in 2018 and 2019. Therefore, the present analysis included data from 2020 onwards.

Prescriber type was reported for all records, so no records were excluded for missing prescriber information. Category‐level missingness was retained and quantified rather than imputed (e.g., PrEP modality “not reported” in 15.6%), given the aggregate, de‐identified nature of the panels. The > 1‐year treatment range field is reported as descriptive markers rather than validated measures of adherence or persistence.

### 2.5 Study Size

The data included the total number of PrEP and PEP dispensing records during the study period; therefore, sample size calculations were not conducted.

### 2.6 Bias

Both monitoring panels relied on pre‐configured and standardized forms, which minimized errors from manual insertions or changes. The databases also provided a data dictionary describing the names of the variables, the variable types (e.g., categorial, numerical, date formats), and their definitions, resulting in consistent terminology [28, 29]. Region and prescriber‐type fields were checked for completeness and internal consistency against the data dictionary during data preparation. As records reflect routinely collected administrative data, differential reporting completeness across regions and services cannot be excluded.

### 2.7 Statistical Analysis

Descriptive statistics were used to characterize the sample. Absolute counts were reported alongside rates and trend estimates. Trends in pharmacist‐prescribed dispensing of HIV prophylactic medications were assessed using joinpoint regression, which tests if the combination of trends provide a statistically better fit to a data series compared with a single‐trend line in time series models [31]. This technique provides the number of joinpoints that are adequate for assessing significant changes over time, enabling the identification of the calendar year where a structural break in temporal trends occurs [32]. Models were fitted to annual observations (calendar years 2020–2025 for PrEP and 2020–2024 for PEP) on the rate per 100 000 adult inhabitants, reflecting the annual dispensing records of the panels and adult population denominators. We employed crude rate as variable type, weighted Bayesian Information Criterion (BIC) as model selection method, and homoscedasticity as variance. Given the limited points per series, the number of joinpoints was constrained to a maximum of one per series, and the optimal number was selected using permutation test with the default error specification. Because each series comprises only five to six annual observations, formal testing for autocorrelation was not employed. We calculated the average annual percent change (AAPC) of pharmacist‐prescribed PrEP and PEP dispensing records per 100 000 adults with 95% confidence intervals (95% CI) per Brazilian region, considering a significance level of 5%. Analyses were conducted using the Joinpoint Regression Program, Version 5.4.0.0 (Statistical Research and Applications Branch, National Cancer Institute).

### 2.8 Ethics Statement and Patient Consent

This study was exempt from ethical approval and patient consent. The research relied exclusively on the analysis of secondary, publicly available, and anonymized data obtained from the “Painel PrEP” and “Painel PEP” databases published by the Brazilian Ministry of Health, preventing linkage to individual patients.

## 3 Results

From 2020 to 2025, 969 536 dispensing records of PrEP were observed in Brazil for all prescribers, of which 27 126 were prescribed by pharmacists (2.80%). Between 2020 and 2024, 664 000 PEP dispensing records were identified considering all prescribers; of those, 13 073 were issued by pharmacists (1.97%). The frequency of pharmacist‐prescribed dispensing records of PrEP increased from 0.22% in 2020 to 5.77% in 2025. Pharmacist‐prescribed PEP dispensing records showed a similar trend, rising from 0.12% in 2020 to 4.59% in 2024 (Table 1).

**TABLE 1:** Frequencies of pharmacist‐prescribed dispensing records of PrEP (2020–2025) and PEP (2020–2024) in Brazil.

PrEP				PEP		
Year	All prescribers	Pharmacist‐prescribed	%	All prescribers	Pharmacist‐prescribed	%
2020	72 376	161	0.22%	95 650	117	0.12%
2021	113 414	983	0.87%	114 107	748	0.66%
2022	183 223	1715	0.94%	129 969	1417	1.09%
2023	234 513	4216	1.80%	149 452	2774	1.86%
2024	189 631	9878	5.21%	174 822	8017	4.59%
2025	176 379	10 173	5.77%	—	—	—
Total	969 536	27 126	2.80%	664 000	13 073	1.97%

*Note:* PEP data were not available for 2025 at the time of the analysis.

Abbreviations: PEP, post‐exposure prophylaxis; PrEP, pre‐exposure prophylaxis.

Most pharmacist‐prescribed PrEP dispensing registries occurred in public healthcare services (*n* = 26 925; 99.2%), under a daily PrEP modality (*n* = 21 686; 79.9%), and with treatment range between first and last dispensing of > 1 year (*n* = 25 908; 95.5%). The majority of patients who received pharmacist‐prescribed PEP were 25–39 years‐old (*n* = 7269; 55.6%), from White/Asian self‐declared race/skin color (*n* = 5781; 44.2%), with ≥ 12 years of educational background (*n* = 6654; 50.9%), and sought PEP after a consented sexual exposure (*n* = 10 139; 77.6%) (Table 2).

**TABLE 2:** Characteristics of pharmacist‐prescribed dispensing records of PrEP (2020–2025) and PEP (2020–2024) in Brazil.

Variables	*n*	%
PrEP		
Total	27 126	100.0
Type of healthcare service		
Public	26 925	99.2
Private	181	0.7
Not reported	20	0.1
PrEP modality		
Daily	21 686	79.9
On demand	1040	3.8
Not reported	4400	16.2
Patients under treatment for > 1 year		
Yes	25 908	95.5
No	1218	4.5
PEP		
Total	13 073	100.0
Age group (years)		
0–14	201	1.5
15–24	2991	22.9
25–39	7269	55.6
40–59	2459	18.8
≥ 60	150	1.1
Not reported	3	0.0
Race/skin color		
White/Asian	5781	44.2
Indigenous	32	0.2
Brown (Brazilian mixed race)	3611	27.6
Black	1332	10.2
Not reported	2317	17.7
Education (years)		
≤ 3	51	0.4
4–7	448	3.4
8–11	3294	25.2
≥ 12	6654	50.9
Not reported	2626	20.1
Type of HIV exposure		
Accident with biological material	2617	20.0
Consented sexual exposure	10 139	77.6
Sexual violence	317	2.4

Abbreviations: PEP, post‐exposure prophylaxis; PrEP, pre‐exposure prophylaxis.

**FIGURE 2:**
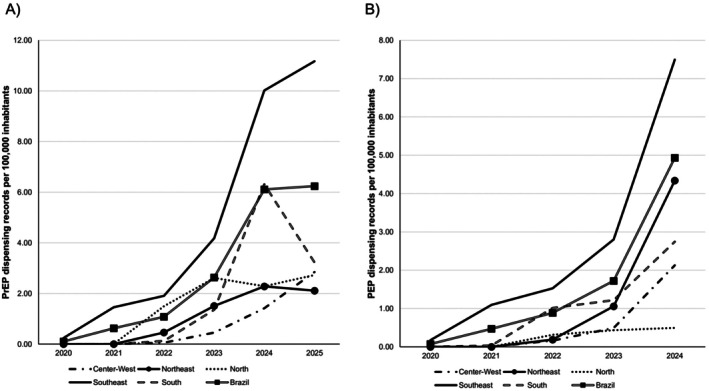
Trends in pharmacist‐prescribed dispensing records of PrEP (2020–2025) (A) and PEP (2020–2024) (B) per 100 000 adult inhabitants per Brazilian region.

The number of pharmacist‐prescribed dispensing records of PrEP per 100 000 adults significantly rose in Brazil over the study period, increasing from 0.10 in 2020 to 6.24 in 2025 (AAPC: 120.80; 95% CI 71.96 to 189.84; *p* < 0.001). The Southeast region recorded the highest absolute rate, increasing from 0.24 in 2020 to 11.17 in 2025 (AAPC: 108.90; 95% CI 48.57 to 193.99; *p* < 0.001). Statistically significant increasing trends were observed in all five regions: Center‐West (AAPC: 514.92; 95% CI 229.50 to 1307.64; *p* < 0.001), Northeast (AAPC: 453.38; 95% CI 170.49 to 1098.35; *p* < 0.001), North (AAPC: 453.56; 95% CI 277.20 to 689.66; *p* < 0.001), and South (AAPC: 618.06; 95% CI 231.18 to 1446.20; *p* < 0.001), with the South showing the steepest relative growth. Structural breaks (joinpoints) were identified in 2022 in the North and in 2023 in the Northeast and Brazil overall, which represent a significant acceleration in pharmacist‐prescribed PrEP dispensing records. Notably, the upward PrEP trajectory showed signs of stabilization in the final year. Between 2024 and 2025, the national pharmacist‐prescribed PrEP dispensing rate remained stable (6.11 to 6.24 per 100 000 adults), corresponding to a 3.0% increase in absolute records (9878 to 10 173). Regional patterns were divergent in this particular period: rates increased in the Center‐West (1.42 to 2.85 per 100 000 adults), North (2.28 to 2.73), and Southeast (10.02 to 11.17), whereas decreases were observed in the Northeast (2.28 to 2.11) and South (6.31 to 3.23; 1522 to 785 records) (Table 3 and Figure 2A).

**TABLE 3:** Number of pharmacist‐prescribed dispensing records per 100 000 adult inhabitants, absolute number of records, and average annual percent change (AAPC) with 95% confidence intervals (95% CI) per Brazilian region for PrEP (2020–2025) and PEP (2020–2024).

Variable	Parameter	Center‐West	Northeast	North	Southeast	South	Brazil
PrEP							
2020	No. per 100 000	0.00	0.00	0.00	0.24	0.00	0.10
	Absolute No.	0	0	0	161	0	161
2021	No. per 100 000	0.00	0.00	0.01	1.45	0.00	0.62
	Absolute No.	0	0	1	982	0	983
2022	No. per 100 000	0.06	0.46	1.49^a^	1.91	0.13	1.08
	Absolute No.	7	193	190^a^	1295	30	1715
2023	No. per 100 000	0.45	1.51^a^	2.61	4.18	1.36	2.63^a^
	Absolute No.	57	638^a^	338	2859	324	4216^a^
2024	No. per 100 000	1.42	2.28	2.28	10.02	6.31	6.11
	Absolute No.	181	975	300	6900	1522	9878
2025	No. per 100 000	2.85	2.11	2.73	11.17	3.23	6.24
	Absolute No.	369	910	364	7745	785	10 173
Total	No. per 100 000	1.93	4.25	6.40	17.79	7.80	10.55
	Absolute No.	614	2716	1193	19 942	2661	27 126
Trend	AAPC (95% CI)	514.92 (229.50 to 1307.64)	453.38 (170.49 to 1098.35)	453.56 (277.20 to 689.66)	108.90 (48.57 to 193.99)	618.06 (231.18 to 1446.20)	120.80 (71.96 to 189.84)
	*p*‐value	**< 0.001**	**< 0.001**	**< 0.001**	**< 0.001**	**< 0.001**	**< 0.001**
PEP							
2020	No. per 100 000	0.00	0.00	0.00	0.17	0.00	0.07
	Absolute No.	0	0	0	117	0	117
2021	No. per 100 000	0.00	0.00	0.00	1.10	0.03	0.47
	Absolute No.	0	0	0	740	8	748
2022	No. per 100 000	0.16	0.19	0.31	1.53	1.01^a^	0.89
	Absolute No.	20	79	40	1038	240^a^	1417
2023	No. per 100 000	0.49	1.06	0.43	2.80	1.22	1.72
	Absolute No.	62	448	56	1917	291	2774
2024	No. per 100 000	2.13	4.34	0.49	7.49	2.75	4.93
	Absolute No.	272	1855	65	5163	662	8017
Total	No. per 100 000	2.78	5.58	1.24	13.09	5.01	8.13
	Absolute No.	354	2382	161	8975	1201	13 073
Trend	AAPC (95% CI)	760.39 (207.52 to 2247.62)	971.59 (317.10 to 2582.75)	532.97 (−14.85 to 4224.75)	134.09 (76.41 to 205.92)	605.98 (−2.83 to 4724.93)	166.62 (68.66 to 313.26)
	*p*‐value	**< 0.001**	**< 0.001**	0.077	**< 0.001**	0.057	**< 0.001**

*Note:* AAPC magnitudes and wide intervals arise from zero baseline rates in 2020–2021 for some regions – for these cases, results should be preferably interpreted with the absolute counts. The bold values are the ones that presented statistical significance.

Abbreviations: AAPC, average annual percent change; No, number; PEP, post‐exposure prophylaxis; PrEP, pre‐exposure prophylaxis.

^a^ Structural break (joinpoint).

The number of pharmacist‐prescribed PEP dispensing records per 100 000 adults presented a similar increasing trend in Brazil: 0.07 in 2020 to 4.93 in 2024 (AAPC: 166.62; 95% CI 68.66 to 313.26; *p* < 0.001). Similarly to PrEP, the Southeast region also had the highest absolute rate of pharmacist‐prescribed PEP during the study period (0.17 in 2020 to 7.49 in 2024; AAPC: 134.09; 95% CI 76.41 to 205.92; *p* < 0.001). Statistically significant upward trends were also observed in the Center‐West (AAPC: 760.39; 95% CI 207.52 to 2247.62; *p* < 0.001) and Northeast (AAPC: 971.59; 95% CI 317.10 to 2582.75; *p* < 0.001). In contrast, trends did not reach statistical significance in the North (AAPC: 532.97; 95% CI −14.85 to 4224.75; *p* = 0.077) or the South (AAPC: 605.98; 95% CI −2.83 to 4724.93; *p* = 0.057), where wide confidence intervals reflect small absolute counts. A structural break (joinpoint) was identified in 2022 in the South (Table 3 and Figure 2B).

## 4 Discussion

This national time series analysis demonstrates a substantial and sustained increase in pharmacist‐prescribed dispensing records of HIV prophylactic medications in Brazil (2020–2025 for PrEP and 2020–2024 for PEP). Following regulatory authorization, pharmacist‐issued prescriptions for both PrEP and PEP rose exponentially, suggesting rapid integration of pharmacists into the multidisciplinary HIV prevention services within the SUS. While pharmacists still account for a relatively small proportion of total prescriptions—2.80% for PrEP and 1.97% for PEP, the frequency of their participation as prescribers has increased more than 26‐fold for PrEP (0.22% in 2020 to 5.77% in 2025) and nearly 38‐fold for PEP since 2020 (0.12% in 2020 to 4.59% in 2024). These findings suggest progressive incorporation of pharmacists into multidisciplinary HIV prevention services within the SUS, reflecting a gradual shift toward broader task‐sharing models in HIV prevention delivery in accordance with international evidence [6, 9, 33, 34].

As an ecological analysis of routinely collected dispensing records, these findings describe temporal trends rather than causal effects; temporal coincidence with regulatory milestones does not establish causation. However, our analysis identified structural breaks (joinpoint) in 2022–2023 varying by region and prophylaxis, which may reflect the process of diffusion of practice models across services. The observed acceleration in prescribing trends coincides with the progressive institutionalization of pharmacist prescribing within the Brazilian HIV response. Initial pharmacist prescribing activities emerged through local institutional protocols implemented in specialized services, particularly in the Southeast region, before later receiving broader regulatory endorsement from professional bodies and the Ministry of Health [11, 12, 13, 14, 15].

Despite representing a minority of total prescriptions, the rapid growth observed suggests that pharmacists are increasingly contributing to HIV prevention delivery within existing care pathways. Previous implementation studies have shown that integrating pharmacists into PrEP and PEP services can improve service accessibility, reduce delays in treatment initiation, and alleviate provider shortages in specialized clinics [6, 7, 8, 9]. In health systems with universal coverage such as Brazil's SUS, task‐sharing approaches that expand prescribing authority among trained healthcare professionals may help address workforce constraints while maintaining integrated models of care [35].

The predominance of dispensing within public healthcare services reflects the central role of the SUS in delivering HIV prevention in Brazil. This finding reflects the structure of the Brazilian HIV response, where biomedical prevention strategies—including PrEP and PEP—are largely implemented through specialized HIV services coordinated within the public health system [10, 36].

Regional differences observed across Brazil may reflect historical inequities in healthcare infrastructure, workforce distribution, and timing of service implementation [18, 37]. For PrEP, statistically significant increases were observed in all five regions, with the Southeast reaching the highest absolute rate and the South, Center‐West, North, and Northeast showing the steepest relative growth from near‐zero baselines in 2020–2021. For PEP, statistically significant increases were observed in the Southeast, Northeast, and Center‐West, whereas trends in the North and South did not reach significance (*p* = 0.077 and *p* = 0.057, respectively). These findings suggest that the expansion of pharmacist prescribing did not occur evenly across Brazilian regions and likely reflects persistent differences in service organization, healthcare infrastructure, and the local uptake of prescribing practices. Alternative explanations should also be considered, including differences in administrative reporting completeness across regions and services and differential timing of protocol implementation, rather than differences in true prescribing activity alone. This interpretation is consistent with prior literature showing that HIV prevention and care in Brazil remain shaped by regional disparities in health system capacity and access, with the Southeast historically concentrating specialized services and prevention delivery [10, 18, 21, 37, 38]. Similar geographic inequalities in access to HIV prevention services have also been described in other settings implementing PrEP scale‐up initiatives, where health workforce distribution and service availability influence prevention coverage [4, 34, 39].

The stabilization of national pharmacist‐prescribed PrEP dispensing—with declines in some regions such as the Northeast and the South—observed between 2024 and 2025 should be interpreted cautiously, as non‐mutually‐exclusive mechanisms may contribute to this pattern. The most recent period of routinely collected administrative databases is frequently provisional and subject to retrospective revision, such that records for the final year are often incompletely captured at the time of extraction and may be adjusted upward as reporting is consolidated [17, 40]. This interpretation is supported by the concurrent decline in total PrEP dispensing across all prescribers in 2025. Moreover, in March 2025 the Federal Council of Medicine obtained a federal court injunction suspending a Federal Council of Pharmacy resolution that regulated pharmacist prescribing of prescription medicines [41, 42]. Although this measure concerned general pharmacist prescribing rather than the protocol‐based prescribing of PrEP and PEP within specialized HIV services, which operates under distinct national clinical protocols, the resulting regulatory uncertainty and surrounding public debate may have had a cautionary influence on prescribing practices during 2025. Given the ecological design and the limited number of annual observations, these explanations remain hypotheses that warrant confirmation as additional years of consolidated data become available.

The patient profile receiving pharmacist‐prescribed PEP—predominantly adults exposed through consensual sexual contact—aligns with epidemiological patterns of HIV exposure reported nationally and internationally [43, 44]. A previous Brazilian cross‐sectional study conducted in an HIV reference center in the Northeast region in 2018 also reported that most PEP prescriptions were issued due to consented sexual intercourse rather than accidental exposure to biological material or sexual violence [23], reinforcing that pharmacist participation occurs within existing prevention healthcare models designed for rapid access following sexual risk events. These findings suggest that integrating pharmacists into specialist HIV services may strengthen prevention responsiveness while maintaining alignment with national clinical workflows and patient populations already prioritized within SUS HIV prevention strategies [15].

Several directions for future evaluation follow from these findings. Pharmacist prescribing and dispensing could be linked to clinical outcomes such as HIV seroconversion, incident sexually transmitted infections, adherence and persistence, and adverse events through record linkage with SUS surveillance and information systems, enabling assessment of effectiveness rather than activity alone. Policy and practice implications could also be derived from these findings, such as investment in protocol‐based training, clinical supervision, and the equitable expansion of pharmacist‐led services in underserved regions such as the North and Center‐West.

This study has limitations. First, the analyses relied on routinely collected administrative dispensing data, which may contain incomplete or misclassified records and do not include clinical outcomes such as adherence, retention, or reductions in HIV incidence. Second, the unit of analysis was dispensing records rather than unique patients, meaning that repeated prescriptions for the same individual may have been counted more than once. Because PrEP is a continuing regimen, increases in PrEP dispensing records may partly reflect repeat dispensing and retention among existing users rather than new initiations; PEP, a single 28‐day course, more closely approximates unique individuals. PrEP findings should therefore be read as trends in dispensing activity rather than confirmed coverage of individuals at risk. Third, while temporal increases in pharmacist prescribing coincided with regulatory and institutional developments, causal relationships between policy changes and prescribing trends cannot be definitively established due to the observational nature of the data. Fourth, given only five to six annual observations per series, formal sensitivity and autocorrelation analyses were not feasible; we therefore report absolute counts alongside rates and interpret estimates from low‐count strata cautiously. Finally, trends were modelled on crude rates with weighted BIC model selection; the very wide confidence intervals for regions with near‐zero baselines warrant cautious interpretation. Nonetheless, the use of nationwide public databases provides a comprehensive overview of real‐world implementation and represents one of the first national evaluations of pharmacist prescribing of HIV prophylaxis in a middle‐income country.

In summary, this nationwide time series analysis demonstrated a substantial increase in pharmacist‐prescribed dispensing records of PrEP (2020–2025) and PEP (2020–2024) in Brazil. Although pharmacists accounted for a relatively small proportion of total prescriptions, their participation as prescribers expanded rapidly during the study period. The Southeast concentrated the largest number of pharmacist‐issued prescriptions and exhibited the highest absolute dispensing rates, while other regions showed heterogeneous growth patterns, suggesting that the implementation of pharmacist prescribing has occurred unevenly across the country. These findings provide early national evidence that pharmacist prescribing is progressively being incorporated into HIV prevention services in Brazil and may represent a complementary workforce strategy to support access to pharmacological prophylaxis within universal health systems. Future research should evaluate clinical outcomes, equity impacts across regions, and the role of pharmacists in prescribing emerging HIV prevention technologies, including long‐acting antiretroviral formulations and differentiated service delivery models.

## Author Contributions

G.M.B.T. contributed to the study design, data analysis, interpretation, and drafting of the original manuscript, and critical revision of the manuscript. D.C.S.A.A. contributed to the supervision, data interpretation, and critical revision of the manuscript. P.M.A. contributed to the supervision, data interpretation, and critical revision of the manuscript. All authors have read and approved the final version of the manuscript.

## Funding

The authors have nothing to report.

## Ethics Statement

This study was exempt from ethical approval. The research relied exclusively on the analysis of secondary, publicly available, and anonymized data obtained from the ‘Painel PrEP’ and ‘Painel PEP’ databases published by the Brazilian Ministry of Health, preventing linkage to individual patients.

## Consent

This study was exempt from patient consent. The research relied exclusively on the analysis of secondary, publicly available, and anonymized data obtained from the ‘Painel PrEP’ and ‘Painel PEP’ databases published by the Brazilian Ministry of Health, preventing linkage to individual patients.

## Conflicts of Interest

The authors declare no conflicts of interest.

## Data Availability

The data will be made available on request. The ‘Painel PrEP’ database is publicly available on https://www.gov.br/aids/pt‐br/indicadores‐epidemiologicos/painel‐de‐monitoramento/painel‐prep. The ‘Painel PEP’ database is publicly available on https://www.gov.br/aids/pt‐br/indicadores‐epidemiologicos/painel‐de‐monitoramento/painel‐pep.
